# Progressive seawater acidification on the Great Barrier Reef continental shelf

**DOI:** 10.1038/s41598-020-75293-1

**Published:** 2020-10-27

**Authors:** Katharina E. Fabricius, Craig Neill, Erik Van Ooijen, Joy N. Smith, Bronte Tilbrook

**Affiliations:** 1 Australian Institute of Marine Science, PMB 3, Townsville, QLD 4810 Australia; 2 CSIRO Oceans and Atmosphere, Castray Esplanade, Battery Point, 7004 Australia; 3 Australian Antarctic Program Partnership, University of Tasmania, Hobart, 7001 Australia

**Keywords:** Biogeochemistry, Environmental sciences, Ocean sciences

## Abstract

Coral reefs are highly sensitive to ocean acidification due to rising atmospheric CO_2_ concentrations. We present 10 years of data (2009–2019) on the long-term trends and sources of variation in the carbon chemistry from two fixed stations in the Australian Great Barrier Reef. Data from the subtropical mid-shelf GBRWIS comprised 3-h instrument records, and those from the tropical coastal NRSYON were monthly seawater samples. Both stations recorded significant variation in seawater CO_2_ fugacity (*f*CO_2_), attributable to seasonal, daytime, temperature and salinity fluctuations. Superimposed over this variation, *f*CO_2_ progressively increased by > 2.0 ± 0.3 µatm year^−1^ at both stations. Seawater temperature and salinity also increased throughout the decade, whereas seawater pH and the saturation state of aragonite declined. The decadal upward *f*CO_2_ trend remained significant in temperature- and salinity-normalised data. Indeed, annual *f*CO_2_ minima are now higher than estimated *f*CO_2_ maxima in the early 1960s, with mean *f*CO_2_ now ~ 28% higher than 60 years ago. Our data indicate that carbonate dissolution from the seafloor is currently unable to buffer the Great Barrier Reef against ocean acidification. This is of great concern for the thousands of coral reefs and other diverse marine ecosystems located in this vast continental shelf system.

## Introduction

Atmospheric carbon dioxide (CO_2_) concentrations are steadily increasing due to human activities, in the present decade at about 2.5 ppm per year^1^. Over a quarter of the rising atmospheric CO_2_ is being taken up by the oceans. This lowers the pH and changes the carbon chemistry in surface seawaters, a process called ocean acidification^2^. Due to human CO_2_ emissions, surface seawater pH is now lower than it has been for more than 800,000 years^3^, and the associated chemical changes are considered to be irreversible on centennial to millennial time scales^4,5^. Many studies have shown that ocean acidification, both in isolation and in combination with global warming, causes profound physiological and ecological changes in marine ecosystems, with far more losers than winners^6–8^. Calcifying marine organisms such as corals and coralline algae are particularly affected, especially during their early life stages, whereas some photosynthetic organisms benefit from the availability of additional inorganic carbon^9,10^.

Rates of changes in seawater carbon chemistry vary substantially across regions, and depend not only on atmospheric CO_2_ concentrations, but also on local physical and biological factors^2,11–13^. In coastal, shelf and marginal seas the variation in seawater carbon chemistry is typically much higher than in the open oceans due to regional metabolic processes (photosynthesis/respiration and calcification). These are sometimes accentuated by terrestrial inputs of carbon, alkalinity, nutrients, freshwater and sediments that directly alter the seawater carbon chemistry and also stimulate biological productivity^14^. In a process called ‘coastal acidification’, acidification may accelerate in areas of eutrophication^2,15^. Coastal CO_2_ concentrations are also affected by the upwelling of CO_2_ rich deeper waters, thermocline shallowing, stratification, freshening and El Niño–Southern Oscillation (ENSO) dynamics. On the other hand, the dissolution of carbonate seafloor sediments can also dampen coastal acidification, depending on carbonate types, grain size and physical condition^16–18^.

High-precision time series data of changes in the carbon chemistry of seawater are available from an increasing number of oceanographic monitoring stations, often above deep waters (> 500 m), but also increasingly so from coastal waters^12,13,19,20^. Many of these data series show increases in seasonally corrected surface CO_2_, at rates similar to that of CO_2_ in the atmosphere^19^. Some stations also show reductions in seawater saturation states of aragonite and calcite, albeit with greater regional differences. In most instances large seasonal variation or decadal oceanographic features require longer observation periods before the detection of significant trends are expected^12^. For example, diel CO_2_ variation of 30–200 µatm day^−1^ and at times over 700 µatm CO_2_ have been recorded for some shallow tropical coastal and shelf marine habitats^21,22^, a variation that is several-fold greater than in the open waters. Given the large temporal and spatial variability and the high ecological and economic value, more long-term carbon chemistry data from coastal and shelf locations are urgently needed.

**Figure 1:**
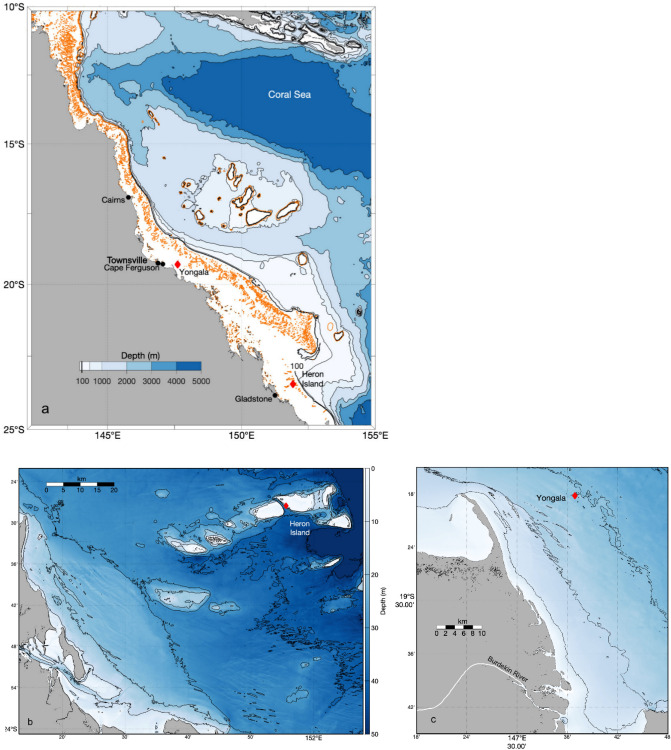
Map of the Great Barrier Reef (orange lines: coral reefs) and the atmospheric gas reference station Cape Ferguson (**a**), and of the oceanographic stations GBRWIS (**b**) and NRSYON (**c**). The blue shading indicates bathymetry, with the thick black line showing the 100-m bathymetry line. The figures were generated using bathymetry data from the 30 m high resolution depth model for the Great Barrier Reef of R. J. Beaman downloaded from Geoscience Australia (https://ecat.ga.gov.au/geonetwork/srv/eng/catalog.search#/metadata/115066), and plotted using Matlab version R2020a software with the M_Map mapping package (version 1.4 m, created by R. Pawlowicz, https://www.eoas.ubc.ca/~rich/map.html).

In this study, we assessed variation and trends in CO_2_ and other proxies for ocean acidification from two sites in the Australian Great Barrier Reef (GBR). The GBR is a shallow carbonate rich continental shelf system extending over 2300 km along the north-eastern Australian coast from 12 to 24° S latitude, and varies in width from 40 to > 200 km (Fig. 1). The GBR is an interesting case study for ocean acidification research, due to its complex hydrodynamic and geomorphological features, and its valuable reef ecosystems. About 5% of the GBR surface is occupied by ~ 3000 coral reefs that are built by corals and other calcifying organisms with a strong dependence on the saturation state of carbonate minerals^8,10,22^. Furthermore much of the seafloor between the coral reefs is covered by biogenic carbonate sediments^23^ with habitats that include mesophotic coral shoals, *Halimeda* mounds, seagrass meadows, sponge gardens and soft bottom ecosystems. Spatial and temporal patterns in the carbon chemistry of the GBR seawater are characterised by substantial cross-shelf gradients, with additional regional features along the coast^24–26^ and in the proximity of coral reefs ^22,27,28^. The surface waters flowing from the Coral Sea onto the GBR shelf are low in nutrient and chlorophyll concentrations, but occasional upwelling onto the shelf break can inject nutrients, total alkalinity and dissolved inorganic carbon into the GBR waters^29,30^. Thirty-five major rivers drain into the GBR lagoon, and their sediment and nutrient loads affect water clarity for months after floods^31,32^. Terrigenous sediments dominate near the mouths of these rivers^33,34^. The ratio of terrigenous to carbonate sediments declines steeply away from the coast, as flood plumes disperse predominantly the fine silts, and storms redistribute terrigenous sediments predominantly within the innermost 15 km of the lagoon^35^.

We present 10 years (2009–2019) of carbon chemistry data together with auxiliary data, from two fixed long-term GBR oceanographic monitoring stations ~ 650 km apart (Fig. 1a, Supplementary Table S1). The first station, GBRWIS^36,37^ (− 23.459° S, 151.927° E), is located in the subtropical southern mid-shelf GBR, at ~ 16 m depth in a channel separating the coral reefs of Wistari Reef from those surrounding Heron Island and ~ 20 km from the edge of the continental shelf (Fig. 1b). The second station, NRSYON^38^ (− 19.305° S, 147.622° E), is a National Reference Station in the tropical central coastal GBR, located at ~ 26 m depth at the Yongala shipwreck near the Burdekin River mouth. We determined the long-term trend and variation in CO_2_, and identified the main physical and chemical drivers of the observed changes. We assessed commonalities and differences of the two stations, and we hindcast changes over the last 60 years. Despite their contrasting settings and substantial seasonal and diel variation, both GBR stations showed significant and rapid rate of increase in CO_2_, hence it is likely that some biological processes in GBR coral reefs are already affected by ocean acidification.

## Results

### GBRWIS station

**Figure 2:**
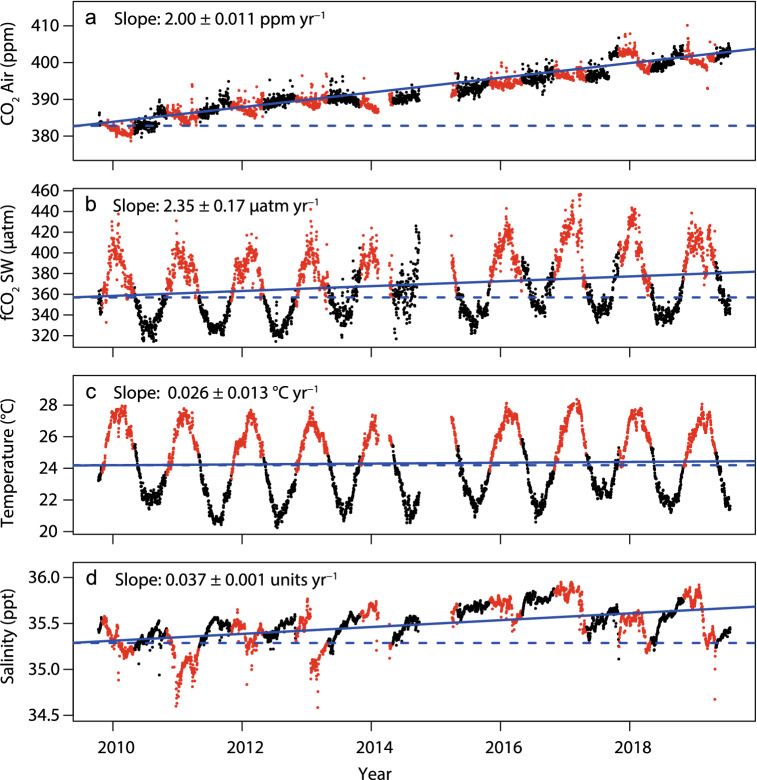
GBRWIS: observed daily averaged data of (**a**) atmospheric CO_2_ (ppm), (**b**) fugacity of CO_2_ in seawater (*f*CO_2_, µatm), (**c**) seawater temperature (°C) and (**d**) salinity. Red points indicate austral summer (November–April, inclusively), black indicate winter (May–October). Solid blue lines show the linear trends, dashed blue lines are horizontal guides. Also shown are the slopes ± 1 standard error.

At GBRWIS, all four instrumental time series showed strong long-term trends, which we refer to in the rest of this paper as the decadal trend. Atmospheric CO_2_ increased at a mean rate of 2.00 ppm year^−1^ ± 0.011 (1 standard error) throughout the 10-year observation period, with only minor seasonal variation (Fig. 2a, Table 1). Mean daily seawater CO_2_ fugacity (*f*CO_2_) increased progressively by 6.4% of its initial value over the decade (Fig. 2b), at a mean rate of 2.35 ± 0.17 µatm year^−1^. The rates of increase in *f*CO_2_ did not differ significantly from that of the atmospheric CO_2_ (GLS on monthly averaged data, N = 109, P = 0.69). Mean daily seawater temperature increased slightly throughout the decade by 0.026 ± 0.013 °C year^−1^, and there was also an upward trend in salinity at a mean rate of 0.037 ± 0.001 units year^−1^ (Fig. 2c,d).

**Table 1:** Atmospheric CO_2_ and surface seawater conditions at the two Great Barrier Reef stations averaged over the years 2009–2019.

	Total mean	Min	Max	Range	Winter mean	5%	95%	Summer mean	5%	95%	Decadal trend (units year^−1^ ± SE)
**Atmospheric CO**_**2**_											
Cape Ferguson Air CO_2_ (ppm)	395.4	384.5	407.8	23.3	394.7	385.7	405.6	395.3	385.3	406.7	2.367 ± 0.0320
Mauna Loa Air CO_2_ (ppm)	398.4	384.4	414.7	30.2	398.6	387.5	410.7	397.3	387.9	407.2	2.389 ± 0.0147
**GBRWIS**											
Air CO_2_ (ppm)	392.4	378.7	410.1	31.4	392.4	383.0	400.8	391.4	381.1	402.4	2.010 ± 0.0634
*f*CO_2_ (µatm)	369.8	314.4	456.5	142.1	339.4	321.2	364.5	405.9	380.7	435.9	1.792 ± 0.3612
Temperature (°C)	24.32	20.24	28.36	8.13	21.65	20.65	22.65	27.04	25.93	27.84	0.0429 ± 0.0127
Salinity	35.49	34.33	35.95	1.62	35.54	35.35	35.77	35.47	34.95	35.86	0.0387 ± 0.0051
**NRSYON**											
DIC (µmol kg^−1^ SW)	1981	1914	2017	103	1991	1975	2005	1966	1922	1990	0.802 ± 0.239
A_T_ (µmol kg^−1^ SW)	2300	2224	2338	113	2304	2273	2324	2293	2237	2319	0.0976 ± 0.256
pH_T_*	8.062	8.003	8.113	0.111	8.096	8.082	8.110	8.035	8.019	8.049	-0.0012 ± 0.0004
Ω_ar_*	3.586	3.230	3.921	0.691	3.455	3.253	3.589	3.735	3.604	3.842	-0.0073 ± 0.0024
*f*CO_2_ (µatm)*	377.1	329.5	442.5	113.0	344.3	331.2	356.2	402.5	388.0	421.0	1.244 ± 0.377
Revelle factor*	9.27	8.90	9.71	0.80	9.42	9.24	9.67	9.09	8.99	9.21	0.011 ± 0.0033
Temperature (°C)	25.77	21.16	29.65	8.49	22.67	21.18	23.80	28.66	27.80	29.44	0.064 ± 0.0269
Salinity	35.29	34.02	36.05	2.02	35.34	34.83	35.60	35.14	34.15	35.62	0.0587 ± 0.0099
DIN (µmol kg^−1^)	0.111	bdl	1.137	1.137	0.123	bdl	0.443	0.106	bdl	0.373	ns
Phosphate (µmol L^−1^)	0.063	bdl	0.24	0.24	0.077	0.02	0.16	0.044	bdl	0.123	ns
Si (µmol L^−1^)	1.29	0.10	3.80	3.7	1.11	0.6	1.73	1.46	0.37	3.09	ns

Total means and ranges: GBRWIS: 3036 daily mean instrument-measured values, NRSYON: 109 monthly mean (depth-averaged) seawater samples. Atmospheric CO_2_ concentrations from Cape Ferguson (https://gaw.kishou.go.jp/search/station#CFA) and from Mauna Loa (sourced from www.esrl.noaa.gov/gmd/ccgg/trends/), and the means and percentiles for the two warmest (January and February) and coldest months (July and August). Also tabled are the estimated slopes (± standard errors) for the decadal trends derived from non-hierarchal GLM analyses of monthly averaged data (the seawater carbonate data corrected for variation with seasons, temperature, salinity and nutrients; Figs. 4, 6; Tables 2, 3; ns = not significant). *Variables calculated from DIC and A_T_ for in situ temperature, pressure, nutrients and salinity, using CO2Sys^61^ with pK1 and pK2 dissociation constants of Dickson and Millero^62^ and KHSO4 dissociation constants of Dickson^63^, total borate of Uppstrom^61^ and the equilibrium constant for HF from Perez and Fraga^62^. *pH*_*T*_ pH at total scale, *Ω*_*ar*_ aragonite saturation state, *fCO*_*2*_ fugacity of seawater CO_2_, *bdl* below detection limit. Measurements of DIC and A_T_ used for CO_2_ system calculations were only available for NRSYON.

Seasonal variation in the daily mean seawater values were high (Fig. 2, Table 1). Daily mean *f*CO_2_ values were on average 67 µatm higher in the two warmest months (January–February: 406 µatm) compared to the two coolest months (July–August: 339 µatm). Seawater temperature varied seasonally by 5.3 °C (27.0 °C versus. 21.7 °C), whereas salinity averaged 35.5 with overall relatively minor systematic seasonal variation, but brief downward spikes that occurred mostly in the wettest summer seasons (Fig. 2d).

**Figure 3:**
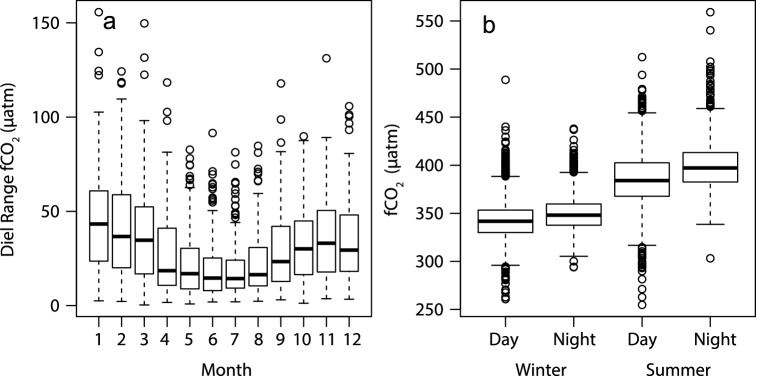
Diel and seasonal variation in *f*CO_2_ at GBRWIS: (**a**) changes in diel ranges of seawater *f*CO_2_ (µatm) across months. (**b**) Mean *f*CO_2_ at day (12:00–16:00 pm) and night (0:00–4:00 am), and in winter (May–Oct) and summer (November–April). Box-whisker plots, with horizontal bars indicating the median, boxes are the 25^th^–75th percentiles, dashed whiskers are 1.5 interquartile ranges, and circles represent outliers (only data from complete years are included).

Diel variation in *f*CO_2_ often exceeded 50 µatm and at times > 100 µatm, particularly in summer (Fig. 3a). Diel fCO_2_ ranges averaged 37 ± 0.71 µatm across the six warmest months (November–April), and 24 ± 0.50 µatm in the six cooler months. Diel ranges in seawater temperature averaged 0.56 ± 0.01 °C and 0.41 ± 0.01 °C in summer and winter, respectively. Overall, mean *f*CO_2_ values were highest during summer nights (midnight to 4 am) averaging 389 µatm (376 µatm in winter nights; Fig. 3b). They were lowest on winter afternoons (noon to 4 pm), averaging 333 ± 0.58 µatm (341 µatm on summer afternoons). Hence mean diel variation in *f*CO_2_ was about 30–50% of the ranges of the mean seasonal variation, and was attributable to changes in temperature and reef metabolism (photosynthetic CO_2_ uptake and night respiration).

**Figure 4:**
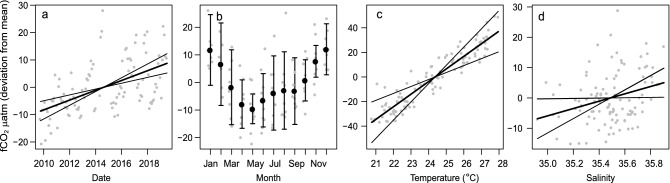
GBRWIS: partial dependency plots showing the changes in estimated monthly mean seawater *f*CO_2_ throughout the decade (**a**), across months (**b**), with temperature (**c**) and with salinity (**d**), while controlling for all other factors (Table 1). y-axis values represent the difference of the predicted *f*CO_2_ response from the mean value (369.8 µatm *f*CO_2_), solid thick lines show the model estimates, i.e., decadal trends (date), seasonal variation (months) and changes related to temperature and salinity. Thin lines and error bars indicate 2 standard errors of the estimates, and grey dots the residuals. Partial dependence plots display model estimates to any predictor value while holding all other predictors constant at their mean (or categorical) values.

To determine the relative strength of the estimated decadal trends and the role of co-varying environmental factors, non-hierarchical GLMs and partial dependence plots were used on monthly averaged data. The decadal upward trends in atmospheric CO_2_, seawater temperature and salinity were significant, with very strong effects of month (representing seasonal variation) for temperature and much weaker effects of month for atmospheric CO_2_ and for salinity (Table 2a). Seawater *f*CO_2_ also displayed a strongly significant decadal upward linear trend, and additionally it varied with temperature, salinity, and month (Fig. 4, Tables 1, 2b). Thus, after adjusting for the variation in seawater temperature, salinity and months using the GLMs, the decadal trend in *f*CO_2_ remained strong and significant (1.792 ± 0.3612 µatm year^−1^, t = 4.960, P < 0.001). The decadal trend was similarly strong for *f*CO_2_-T, i.e., after temperature normalisation to remove the influence of seawater temperature on the *f*CO_2_ trend following Takahashi et al.^39^, the decadal *f*CO_2_-T trend was 1.828 ± 0.628 µatm year^−1^ (t = 5.009, P < 0.001). The strongest predictor for variation in both *f*CO_2_ and *f*CO_2_-T at GBRWIS was the decadal linear trend (Table 2b).

**Table 2:** GBRWIS: factors related to the observed variation in (a) atmospheric CO_2,_ seawater temperature and salinity, and in (b) *f*CO_2_ (Fig. 4) and *f*CO_2_ normalised to the local mean temperature (24.34 °C)^39^.

(a)	df	Atmospheric CO_2_		Temperature		Salinity	
		F	P	F	P	F	P
Decadal trend	1	1881.1	< 0.001	11.42	0.001	58.43	< 0.001
Month	11	2.789	0.0034	267.5	< 0.001	3.527	< 0.001

(b)	df	*f*CO_2_		*f*CO_2_-T			
		F	P	F	P		
Decadal trend	1	24.6	< 0.001	25.10	< 0.001		
Month	11	4.148	< 0.001	3.818	< 0.001		
Temperature	1	19.9	< 0.001	5.283	0.024		
Salinity	1	4.399	0.039	2.959	0.089		

Non-hierarchal generalized linear model (GLM) analysis of monthly mean data (N = 109 months).

### NRSYON station

**Figure 5:**
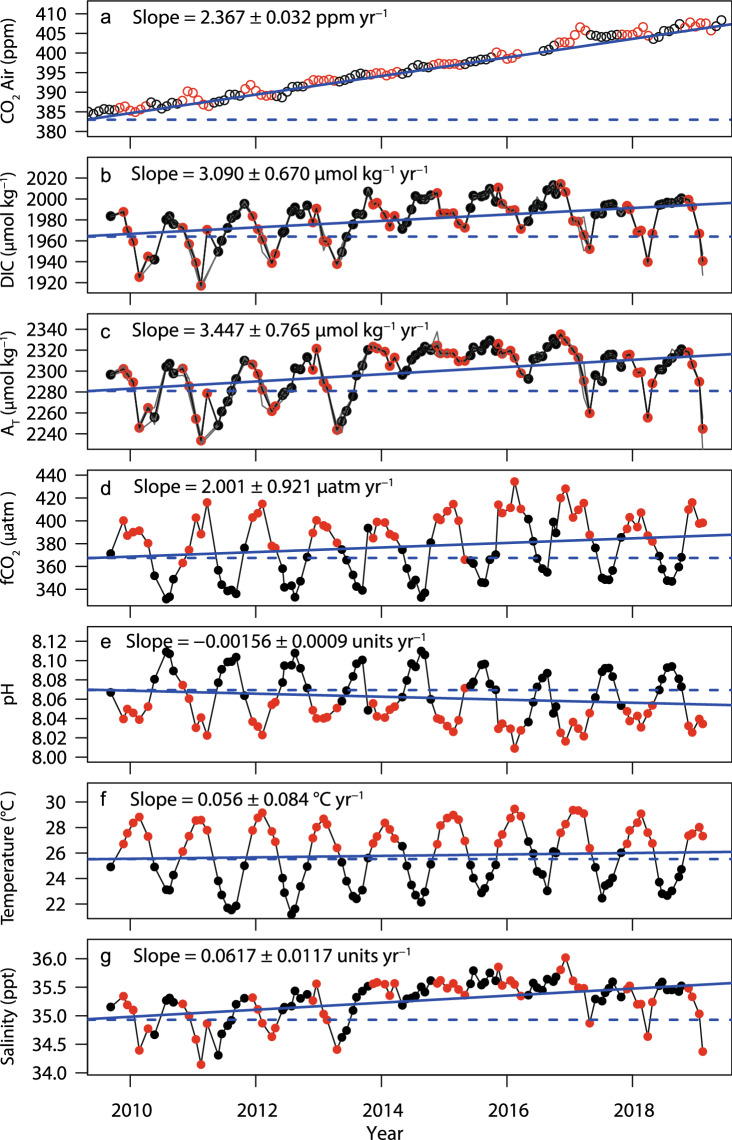
NRSYON: observed atmospheric CO_2_ and seawater carbon chemistry values from 2009 to 2019. (**a**) Atmospheric CO_2_ concentrations at the Cape Ferguson greenhouse gas monitoring station. (**b**) To (**g**): dissolved inorganic carbon (DIC), total alkalinity (A_T_), temperature and salinity (all measured); *f*CO_2_ and pH (calculated). Points are depth-averaged monthly seawater samples; red points indicate summer (November–April); black are winter (May–October). In (**b**,**c**), the thin grey lines show the four sampling depths (0, 10, 20, 26 m). Straight solid blue lines are the linear trends, dashed blue lines are horizontal guides. Also shown are slopes of daily averaged data, unadjusted for changes in temperature and salinity.

Atmospheric CO_2_ concentrations at the Cape Ferguson greenhouse gas station increased near-monotonically from 353 ppm in June 1991 to 408 ppm in July 2019. Between 2009 and 2019, the mean rate of increase was 2.37 ± 0.03 ppm CO_2_ year^−1^ (Fig. 5a, Table 1).

The NRSYON seawater samples showed no overall significant differences across the four sampling depths (Fig. 5b,c), despite some individual samples showing depth differences that suggested occasional weak stratification especially in summer. Based on the absence of systematic depths differences, all data were averaged across depths within sampling months for the following analyses (N = 107).

NRSYON data displayed strong seasonal variation (Fig. 5b–g; Table 1). *f*CO_2_ values ranged over 113 µatm, being on average 58 µatm higher in the warmest vs the coolest months (402.5 µatm vs 344.3 µatm). Ω_ar_ was also higher (3.76 vs 3.45). Mean seawater temperatures were 6 °C higher during the two warmest compared to the two coolest months (January/February: 28.7 °C, July/August: 22.7 °C), while salinity and pH_T_ were lower in the warmest versus the coolest months. Mean concentrations of dissolved inorganic nitrogen, total phosphorus and silicate were relatively low, however variation was high (Table 1).

Superimposed over the seasonal variation, many of the NRSYON data showed strong decadal trends (Fig. 5b–g). Measured DIC increased by 3.09 ± 0.67 µmol kg^−1^ SW year^−1^. Calculated *f*CO_2_ increased on average by 2.00 ± 0.92 µatm year^−1^, at a rate that was not significantly different from that of the atmospheric CO_2_ trend at nearby Cape Ferguson (2.37 ± 0.03 ppm CO_2_ year^−1^; GLS, P = 0.69). Calculated pH_T_ declined by 0.00156 ± 0.0009 units, and Ω_ar_ by 0.0044 ± 0.0048 units year^−1^. Seawater temperature and salinity also increased over the decade (Fig. 5f–g).

**Figure 6:**
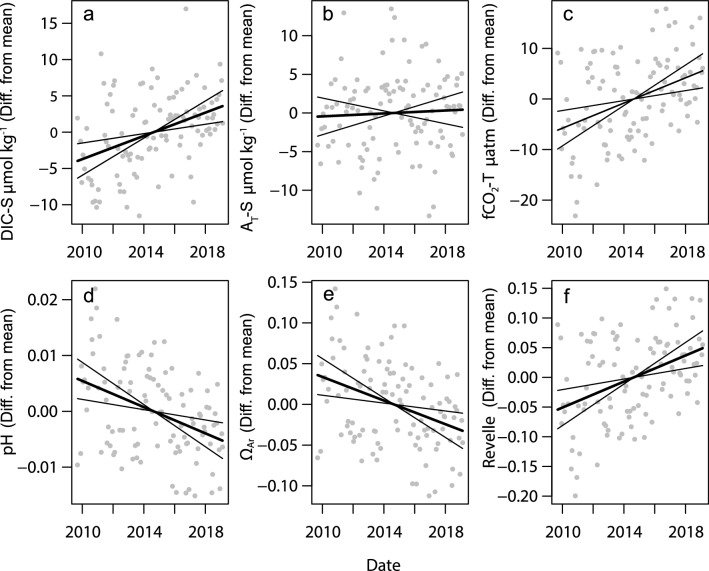
NRSYON: partial dependence plots showing the changes in estimated depth-averaged DIC and A_T_ (**a**,**b**), and the other carbon chemistry variables (**c**–**f**) related to decadal trends (date). Trend estimates of are controlled for changes across months and in temperature, salinity and nutrients (Table 3, Supplementary Fig. S1). Values on the y-axis represent differences from the mean values (Table 1). Solid thick lines are the model estimates, thin lines are 2 standard errors of the estimates, and grey dots the residuals.

The statistical analyses showed the decadal upward trends in temperature and salinity to be significant (P < 0.05), albeit only marginally so for temperature (non-hierarchal GLM analyses, also accounting for seasonal variation; Table 3). For DIC, *f*CO_2_, pH and Ω_ar_, but not for A_T_, the decadal trends remained significant after accounting for seasonal variation (months) and changes in temperature, salinity and nutrients (dissolved inorganic nitrogen, phosphorus and silicate) (Fig. 6, Supplementary Fig. S1a,b, Table 3). For DIC and A_T_, the strongest predictor was salinity, with weaker but significant effects from DIN and month. Calculated *f*CO_2_, pH, Ω_ar_ and the Revelle factor were most strongly related to temperature, followed by the decadal trend and silicate. After normalisation to mean salinity values following Friis et al.^40^, the decadal trend in DIC-S became weaker and for A_T_-S it became insignificant (Supplementary Table S1, Fig. 1C). However, salinity remained a significant predictor, suggesting slight over-compensation by the normalisation. Normalisation of *f*CO_2_ to mean temperature also showed that the decadal trend remained significant at 1.259 µatm year^−1^ ± 0.3696, P < 0.001 (also accounting for changes in salinity, nutrients, and months; Supplementary Table S1). For the nutrients, silicate was significantly associated with all carbon variables except for A_T_. DIN was significantly associated with DIC and DIC-S and marginally with A_T_ and A_T_-S. Phosphate was only weakly associated with DIC and DIC-S.

**Table 3:** NRSYON: environmental and temporal factors associated with changes in seawater carbon chemistry variables.

(a)	df	Temperature		Salinity		DIC		A_T_	
		F	P	F	P	F	P	F	P
Decadal trend	1	5.678	0.037	34.97	< 0.001	11.21	0.001	0.145	0.704
Month	11	86.53	< 0.001	4.947	< 0.001	4.517	< 0.001	3.859	< 0.001
Temperature	1					2.119	0.149	6.673	0.011
Salinity	1					361.2	< 0.001	514.1	< 0.001
DIN	1					7.116	0.009	4.151	0.045
Phosphate	1					4.625	0.034	1.118	0.293
Silicate	1					0.971	0.327	4.671	0.033

(b)	df	*f*CO_2_		pH		Ω_ar_		Revelle Factor	
		F	P	F	P	F	P	F	P
Decadal trend	1	10.92	0.001	10.80	0.002	8.994	0.004	11.30	0.001
Month	11	1.744	0.076	1.829	0.061	1.812	0.063	1.865	0.055
Temperature	1	50.15	< 0.001	55.64	0.000	47.61	< 0.001	31.78	< 0.001
Salinity	1	6.161	0.015	0.272	0.604	25.08	< 0.001	4.973	0.028
DIN	1	1.175	0.281	0.560	0.456	0.154	0.696	0.825	0.366
Phosphate	1	2.416	0.124	1.885	0.173	1.016	0.316	2.362	0.128
Silicate	1	11.16	0.001	11.49	0.001	12.63	0.001	10.43	0.002

Non-hierarchal generalized linear model (GLM) analysis of monthly depth-averaged data (N = 107 months). Tabled are the decadal trend and variation due to seasons (Month) for seawater temperature and salinity, and the decadal trend, seasonality and environmental predictors (seawater temperature, salinity and nutrients) for selected carbon chemistry variables: measured DIC and A_T_, and calculated *f*CO_2_, pH, and the Revelle factor (Fig. 6, Supplementary Figure S1a,b). Salinity and Temperature normalised values are shown in Supplementary Table S1 and Supplementary Fig. S1c.

## Discussion

### Long-term trends

Two long-term monitoring stations in the GBR, 650 km apart in very different environmental settings, display significant upward trends in their near-surface seawater carbon chemistry. The two GBR stations show very similar rates of *f*CO_2_ increase, at ~ 2.0 ± 0.3 µatm year^−1^, additional to substantial seasonal and moderate diel variation. These trends are quite similar to rates documented for open ocean regions^12,13,19,41,42^. For example, pCO_2_ at the pelagic Hawaiian Station ALOHA increased at 2.5 ± 0.1 ppm year^−1^ between 1988 and 2002, albeit with much weaker (~ 20 µatm) seasonal variation^43^. Similarly, two oceanic long-term monitoring stations, WHOTS in the subtropical North Pacific and Stratus in the South Pacific gyre, show pCO_2_ trends of 1.9 ± 0.3 and 1.6 ± 0.3 µatm year^−1^, respectively. At the North Atlantic Ocean ESTOC site (offshore from the Canary Islands), *f*CO_2_ increased by 1.55 ± 0.43 µatm year^−1^ between 1995 and 2004, which the authors attributed to atmospheric forcing together with large-scale oceanic and climatic features^44^. For pH, the NRSYON trend (− 0.0016 ± 0.0009 pH units year^−1^) was also similar to other published offshore and pelagic long-term seawater carbon monitoring stations (− 0.0016 to − 0.0018 pH units year^−1^), in agreement with those expected under air-sea CO_2_ equilibration (− 0.0016 to − 0.0018 pH units year^−1^)^19,44^.

The similarity in the decadal CO_2_ trends at both GBR stations to the atmospheric CO_2_ changes suggests these CO_2_ trends are largely determined by atmospheric forcing, as also found at the open ocean sites. This assumes there has been no significant reorganisation of the biological and physical processes influencing surface water carbon chemistry over the decade of measurements at our two sites. NRSYON is strongly influenced by coastal processes and is close to a major river outflow, but ~ 100 km from the shelf edge, whereas the GBRWIS site is located next to a coral reef but 68 km off the coast and within 20 km of the shelf edge and the open-ocean waters of the Coral Sea. The buffering capacity by the carbonate sediments that cover most of the seafloor in the GBR was clearly insufficient to prevent the rapid acidification of the water body near these two stations for the 10-year duration of the measurements. Based on this similarity we suggest the observed decadal trends are unlikely to be exclusive to the two sites and reflect the trend over a broad region of the central and South GBR.

Seawater temperature and salinity also showed significant upward trends over time at the two GBR stations, consistent with regional oceanographic and climatological features. The physical oceanography of the GBR is dominated by the inflow of Coral Sea water which bifurcates offshore between about 15 and 20° S, leading to predominantly southward-bound offshore currents, and northward bound inshore counter currents in the southern and central GBR^45^. Sea surface temperatures are increasing over the whole GBR region with changes in Coral Sea source waters of 0.1–0.2 °C per decade^46^. The variable salinity trend is consistent with temporal and regional variation in monsoonal rainfall and river flows, and also reflects slight salinity increases in Coral Sea waters^41^. The climate of Northeast Australia is additionally influenced by ENSO cycles, which typically have a 2–7 years duration. At both sites, El Niño (La Niña) events were associated with anomalously high (low) salinities compared to the long-term trend. La Niña conditions with increased rainfall were present from mid-2010 until about March 2012^47^. By mid-2014, a moderate to strong El Niño with low rainfall emerged and persisted until mid-2016, followed by neutral conditions^48^. At GBRWIS, the downward spikes in salinity coincided with large rainfall events in 2010/11, 2013 and 2019. The coastal NRSYON site had a greater variability and trend in salinity than GBRWIS, likely attributable to its greater exposure to coastal inflows, and the influence of net evaporation or precipitation in shallower water. The Burdekin River had large discharges and northward plume flows in the summers of 2007/08 to 2011/12 and 2018 and 2019 with seasonally low salinities at NRSYON, and low discharges from 2012/13 to 2017/18 that corresponded with higher salinities in the summer wet season at the site.

Both temperature and salinity play an important role in determining changes in the seawater carbon chemistry, directly as temperature determines the solubility of CO_2_ and salinity affects A_T_ and DIC, and indirectly as biotic metabolism accelerates with temperature. Total alkalinity at NRSYON was strongly related to salinity, with prolonged reductions in years with high river flows. The decadal increase in temperature and salinity, and resulting changes in A_T_ and DIC at NRSYON will cause an increase in *f*CO_2_ and decrease in pH and Ω_ar_. However, the decadal upward trend in *f*CO_2_ remained significant after normalisation to a mean temperature at both sites, and also after statistically adjusting for the effects of rising temperatures and salinity. This again confirms the strong role of atmospheric forcing in driving the trends in seawater CO_2_ chemistry over the duration of our study. In addition to the decadal trends at both sites, there is substantial seasonal variation in the carbon chemistry due to seasonal temperature fluctuations and biological activity. Longer term observations through multiple ENSO events are needed to resolve the combined influences of local and large-scale controls on the rate of ocean acidification in the GBR more clearly.

**Figure 7:**
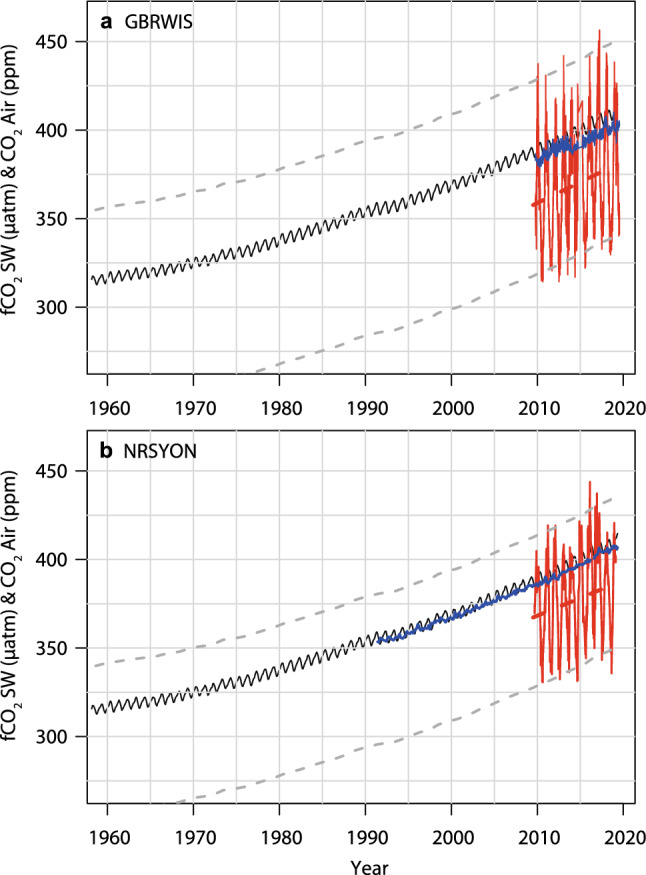
Observed changes in atmospheric CO_2_ concentrations at Mauna Loa monitoring station^1^ (solid black line), and at GBRWIS since 2009 (**a**, blue line) and Cape Ferguson since 1991 (**b**, blue line; sourced from https://gaw.kishou.go.jp/search/station#CFA). Red lines: observed seawater *f*CO_2_ and their linear fits at GBRWIS (**a**) and NRSYON **(b**). Grey dashed lines are back-projections of observed seawater *f*CO_2_ envelopes. Also shown are slopes of the data prior to correction for variation in temperature, salinity, seasonality, and nutrients.

We hindcasted *f*CO_2_ conditions in the GBR over the last 60 years, when the longest existing greenhouse gas records commenced at Mauna Loa, Hawaii (since 1958; www.esrl.noaa.gov/gmd/ccgg/trends/). The decadal trends in the atmospheric CO_2_ time series from both GBRWIS and Cape Ferguson closely matched the winter values of Mauna Loa (Fig. 7). From 2009 to 2019, atmospheric CO_2_ at Mauna Loa increased by ~ 2.2 ± 0.01 ppm year^−1^ (NOAA 2019), at GBRWIS by 2.00 ± 0.011 ppm year^−1^, and at Cape Ferguson by 2.37 ± 0.03 ppm year^−1^ (Figs. 2, 5, 7). The slopes of seawater *f*CO_2_ at GBRWIS and NRSYON over the same time period were also statistically similar, increasing at 2.35 ± 0.17 and 2.00 ± 0.92 µatm year^−1^ respectively, with an offset by − 26 ppm for GBRWIS and − 13 µatm for NRSYON compared with their atmospheric CO_2_ values. Our data suggest that today’s *f*CO_2_ minima are likely above the maxima these two sites would have experienced in the early 1960s (Fig. 7). At NRSYYON, the current (2009–2919) average range of winter to summer *f*CO_2_ is 344.3–402.5 µatm (Table 1) and extrapolation back to 1958 gives a range of about 261–319 µatm, i.e., the current envelope is outside than from 50 years ago. At GBRWIS, only *f*CO_2_ and no other systematically sampled carbon chemistry data are available over the 10-year sampling period, however the present day average winter-summer range of *f*CO_2_ (339.4–405.9 µatm) is also probably outside the average seasonal range in the early 1960s of 333–250 µatm (Fig. 7). Our hindcasts assume that *f*CO_2_ at the GBR sites have tracked the atmospheric increase at Mauna Loa, and that GBR seawater A_T_ has remained similar since 1958. The observed long-term changes in temperature and salinity are too small to alter this result. The increasing Revelle Factor with time due to surface water CO_2_ uptake has likely amplified rather than diminished the seasonal signal today relative to that of the 1960s. Factors such as a widespread decline in net calcification on the reef since the 1960s may have altered the *f*CO_2_ ranges by changing A_T_ and DIC concentrations, however few data from before 2009 exist to determine if this is has occurred. Therefore, despite the highly dynamic nature of both stations, their *f*CO_2_ concentrations are now likely about 28% higher than in the 1960s and outside the envelope that both locations would have experienced only 60 years ago. These findings match those from open ocean subtropical and subarctic moorings, where today’s surface seawater chemistry conditions are also largely or entirely outside of the bounds of preindustrial values throughout the year^20^.

At NRSYON, average Ω_ar_ would likely have been ~ 0.33 units higher at ~ 3.92 in the 1960s than it is today at 3.59. Similarly, today’s pH at NRSYON averages 8.062, and has likely dropped by ~ 0.07 units, so pH may have averaged around 8.13 at this site in the early 1960s. These estimates add further confidence to earlier reconstructions of changes in the seawater carbon chemistry in the Australian region from salinity, atmospheric CO_2_ and temperature data^37^, which suggested an Australia-wide mean decline in Ω_ar_ by 0.48 units and in pH by 0.09 units since 1870, in response to increasing oceanic uptake of atmospheric CO_2_.

### Variation in seawater *f*CO_2_, and the role of environmental drivers

Surface seawater carbon concentrations showed substantial seasonal variation compared to the atmospheric CO_2_, likely attributable to seasonal changes in temperature and salinity, to benthic and pelagic production, as well as to differences in source water bodies^25^. However, observed ranges in *f*CO_2_ at the two GBR stations were moderate compared to many other coastal stations^15^. Total ranges were 143 µatm at GBRWIS and 113 µatm at NRSYON, while seasonal ranges averaged 67 µatm at GBRWIS and 58 µatm at NRSYON. Diel ranges were only assessed at GBRWIS, where they averaged 40 µatm in summer and 24 µatm in winter. In comparison, at moorings around O'ahu, Hawai'i (2008–2016), the coastal pCO_2_ station in Kaneohe Bay behind a strongly calcifying shallow coral reef with slow turnover and strong terrestrial influences displayed a maximum CO_2_ range of 950 µatm pCO_2_, while two other Hawai'ian stations with less calcification exhibited surface water pCO_2_ variability ranging from 198 to 240 µatm ^21^. Mean diurnal variability at Kaneohe Bay at 192 µatm was also 4–5 times greater than at GBRWIS. In Hawai'i as in the GBR, the seasonal seawater CO_2_ dynamics were opposite to those in the atmosphere, with highest seawater CO_2_ values in summer and lowest values in winter.

Seasonal variation was greater on the mid-shelf GBRWIS than at the coastal NRSYON (Table 2), and there was a larger mean gradient between seawater and atmospheric CO_2_ at GBRWIS compared to NRSYON (26 vs 13 ppm). A comparison between the two stations needs to be done with caution: at GBRWIS the *f*CO_2_ was directly measured by instruments day and night and in all weather conditions but only at about 0.5 m water depth. At NRSYON, *f*CO_2_ was calculated from seawater DIC and A_T_ sampled at four depths through the 26 m water column and samples were only collected during daylight and not in very windy conditions. The subtropical GBRWIS also experiences slightly greater seasonal variation in daylength and temperature compared to the tropical NRSYON, likely contributing to its greater variability. Furthermore GBRWIS is located near shallow and actively calcifying coral reefs, while NRSYON is located near a major river in ~ 26 m deep turbid water without shallow reef structures nearby, and light on the seafloor is low^49^. Consequently, the greater diel fluctuations in *f*CO_2_ in summer than winter at GBRWIS are likely attributable to daytime photosynthesis and night respiration being greater at the higher temperatures and higher PAR in summer. Considering all these factors, it appears the biological processes on the coral reefs had a greater effect on the local carbon chemistry around GBRWIS than did the more variable salinity, nutrients, sediments and inter-reefal benthos near the Burdekin River at NRSYON.

Seawater nutrient concentrations were positively related to some of the carbon chemistry variables at NRSYON (Supplementary Fig. S1, Table 2). However mean concentrations of dissolved macronutrient are typically low in the tropics except in waters exposed to terrestrial runoff and upwelling, with maximum observed concentrations of phosphate and DIN of 0.24 and 3.8 mmol/kg, respectively (Table 1). River nutrient load data are only available on a yearly basis, so could not be included into the models. However, at this site macronutrient availability did not appear to be a dominant driver of the CO_2_ variation on seasonal scales. Overall, concentrations of alkalinity and DIC appear typically lower in Burdekin River discharge waters than in seawater, but they are higher in alkalinity than in DIC^50^. Due to the sparsity of relevant river data and complex hydrodynamics, it remains unclear to what extent rivers affect the alkalinity and inorganic carbon concentrations of GBR ecosystems, either directly through their DIC, alkalinity and nutrient loads, or indirectly via stimulation of biological processes.

## Conclusions

We have shown that the carbon chemistry at the two GBR stations has changed rapidly over the last decade, with decadal trends that are consistent across locations. The close tracking of atmospheric and aquatic CO_2_ in the GBR suggests that its *f*CO_2_ has increased by about 28% since the early 1960s, and that projections of environmental conditions from large scale-carbon cycle models may be used to track changes in the carbon chemistry within the GBR. Our data demonstrate that ocean acidification is not merely a concern for the future of the GBR. Rather, it is already part of its rapidly changing chemical and physical conditions in the Anthropocene. The potential buffering capacity of the surrounding shallow carbonate seafloor in this continental shelf system, and the dynamics caused by the metabolism on its coral reefs and its large rivers, are ineffective in protecting this vast system from ocean acidification based on the ~ 10 years of data we analysed. Seawater *f*CO_2_ concentrations in the GBR are now outside the envelope the GBR experienced in the 1960s, and trends are expected to continue and even accelerate throughout this century, due to the increasing Revelle factor^51,52^.

The implications of this pervasive and rapid change in the concentration of a biologically highly active parameter together with seawater warming, will greatly depend on the location of potential refugia from ocean acidification (if any), and CO_2_ emission trajectories. A large number of experimental and in situ studies around volcanic CO_2_ seeps have demonstrated the profound implications of ocean acidification for the ecological integrity of coral reefs, including reduced coral calcification, coral biodiversity, coral recruitment, structural complexity, reef accretion rates, and coralline algal cover, and increasing seagrass and macroalgal cover^8,9,53^. Today’s mean Ω_ar_ at NRSYON (3.59, down from a predicted historical mean value of ~ 3.92 in the 1960s) is near the measured tipping point of Ω_ar_ = 3.5 to 3.6 for the GBR, at which level crustose coralline algae and the densities of coral juveniles steeply decline and macroalgal cover increases^10^. Eyre, et al. ^16^ also concluded that the GBR is close to a tipping point when dissolution of carbonate sediments on coral reefs exceeds its generation. Although our data do not support the notion that the carbon chemistry trends at the two sites are modified as yet by net dissolution, longer term records and more measurements in the proximity of benthos are needed to assess this concern. Indeed, the contrasting setting of these stations is valuable in assessing whether or not the GBR is tipping towards conditions of net dissolution. Our study involved only two sites in the south and central GBR, and more data are needed from along its 2300 km length to characterize in greater detail how conditions are changing over time in the broad range of GBR environments (coral reefs, coastal regions including mangroves and estuaries, seagrass meadows and inter-reef regions).

The ongoing support for a global network of *f*CO_2_ and carbon chemistry monitoring sites will remain essential to improve predictions of regional seawater conditions and their drivers. Our data complement other studies^54,55^ that show that ocean acidification driven by CO_2_ uptake from the atmosphere, together with the rapid increases in global temperatures, is an immediate concern for the persistence of healthy ecosystems in the GBR, rather than a future problem. The data further contribute to the resounding scientific call for immediate and drastic global cuts in CO_2_ emissions, combined with strong local management action, to protect coral reefs into the future.

## Methods

### GBRWIS station

Concentrations of CO_2_ in the air (ppm) and surface seawater fugacity of CO_2_ (*f*CO_2_, µatm), temperature (°C) and salinity data were sourced from GBRWIS Station, located in 16 m water depth in a channel between Heron and Wistari Reef, near the eastern side of Heron Island, southern GBR on the mid-shelf (− 23.459° S, 151.927°E; Fig. 1a,b). This CO_2_ Acidification Mooring surface buoy is part of the Australian Integrated Marine Observing System (IMOS) National Mooring Network, maintained by CSIRO, Hobart^36,37^, with newly calibrated sensors deployed every ~ 6 months. Surface water and atmospheric CO_2_ are measured on either a 2- or 3-h measurement cycle using a Battelle Seaology pCO_2_ monitoring system (MApCO_2_), while temperature and salinity of the surface seawater and the equilibrator are measured with a Seabird Scientific SBE16plusV2. The CO_2_ measurement uses a bubble equilibrator with an intake at about 0.5 m depth. Air from the equilibrator headspace is circulated through a LI-COR 820 non-dispersive infrared detector (NDIR)^56,57^ and a two-point calibration is automatically conducted before every seawater and air CO_2_ measurement, using a zero CO_2_ gas (air cycled through a soda lime chamber to remove CO_2_, a nafion drier surrounded by silica gel to remove water vapour) and a CO_2_-in-air span gas (500–550 µmol/mol, prepared by the NOAA Earth Systems Research Laboratory and calibrated on the WMO X2007 scale with a standard deviation of 0.06 µmol/mol; https://www.esrl.noaa.gov/gmd/ccl/airstandard.html). Data from 9/10/2009 up to 12/03/2019 were quality-controlled delayed-mode sourced from the Australian Ocean Data Network (https://portal.aodn.org.au/search), those to 13/10/2019 were near-real-time auto quality-controlled data stream. Data were filtered to exclude values flagged as questionable or bad^37^. For the trend analysis, data points with salinity < 34 PSU were removed (51 of 36,590 points) before creating daily averages. Days with < 7 samples per day were also removed, and the remaining 3242 daily means were averaged to monthly values. Removal of three incomplete months created 109 monthly values.

### NRSYON station

Data of total alkalinity and total dissolved organic carbon, nutrients and salinity, were sourced from bottle samples taken at NRSYON, an IMOS National Reference Station at Yongala shipwreck in the central coastal GBR (− 19.305° S, 147.622° E ^38^). NRSYON is located in 26 m water depth, ~ 35 km downstream from the Burdekin River mouth (Fig. 1a,c). The Burdekin River is the largest river entering into the GBR, with annual mean discharges of 7600 GL freshwater, 4.7 million tons of fine sediments, and 9000 tons of particulate nitrogen^33^, causing significant intra- and inter-annual variability in salinity, nutrients and turbidity^31^. Samples were collected by AIMS approximately monthly from September 2009 to February 2019. Water was collected in Niskin bottles from 0, 10, 20 and 26 m depth from a small boat, typically during calm conditions and only during day times. For the analysis of total dissolved inorganic carbon concentrations (DIC [µmol kg^−1^ seawater]) and total alkalinity (A_T_ [µmol kg^−1^ seawater]), samples were drawn from the Niskin bottles into 250 ml Schott bottles using silicone tubing to avoid bubble formation and minimize headspace, preserved with 125 µl of saturated HgCl_2_, stored at room temperature in darkness, and sent to CSIRO Hobart for analysis. Sampling and analysis followed the IMOS National Reference Stations protocol^58^. A_T_ was measured by open cell titration using a Metrohm Titrando and followed standard operating procedures^59^. DIC was measured by coulometric titration with a SOMMA instrument^60^, and the salinity of the samples was measured by a Seabird conductivity cell associated with the SOMMA (metadata: https://catalogue-imos.aodn.org.au/geonetwork/srv/eng/metadata.show?uuid=fa93c66e-0e56-7e1d-e043-08114f8c1b76). Precision and reproducibility for DIC and A_T_ was estimated from measurements of seawater reference material (Dickson laboratory, Scripps Institution of Oceanography) and of duplicate samples. Carbon data are available through https://portal.aodn.org.au/search. Samples for nutrients, including dissolved inorganic nitrogen (NH_4_^+^, NO_3_^−^/NO_2_), total silicate and total phosphorus were immediately filtered, and measured spectrophotometrically (metadata: https://catalogue-imos.aodn.org.au/geonetwork/srv/eng/metadata.show?uuid=fa93c66e-0e56-7e1d-e043-08114f8c1b76). Temperature data were compiled from data loggers maintained at the station^37^.

Data of DIC, A_T_, temperature, salinity and nutrients were used to compute the other seawater carbon chemistry variables (aragonite saturation state Ω_ar_, pH at the total scale (pH_T_), fugacity and partial pressure of CO_2_ (*f*CO_2_ and *p*CO_2_) and the Revelle factor) for observed in situ temperature, pressure, nutrients and salinity, using CO2Sys^61^, with pK1 and pK2 dissociation constants of Dickson and Millero^62^ and KHSO_4_ dissociation constants of Dickson^63^, total borate of Uppstrom^61^ and the equilibrium constant for HF from Perez and Fraga^59^. For consistency with the GBRWIS *f*CO_2_ data, our reporting of NRSYON CO_2_ trends focused on *f*CO_2_. Data points with salinity < 34 PSU were removed for the trend analyses (12 of 432 points). Despite significant depth differences on several days, there were no significant overall differences across the four sampling depths, hence all data were depth-averaged to monthly mean values (N = 107). Data on DIC, AT and *f*CO_2_ data are first presented as observed at in situ temperature and salinity values.

### Greenhouse gas monitoring station Cape Ferguson

Regional data of atmospheric CO_2_ concentrations as dry mole fractions were sourced from the greenhouse gas monitoring station Cape Ferguson (− 19.2773° S, 147.0587° E, central GBR, ~ 30 km from NRSYON; Fig. 1a). Since May 1991, triplicate discrete air flask samples have been collected monthly at this AIMS jetty, during times when winds were onshore (60°–160°) to minimise land influences_._ Samples are analysed by CSIRO Oceans and Atmosphere, Climate Science Centre-GASLAB, and provided through the World Data Centre of Greenhouse gases, https://gaw.kishou.go.jp/search/station#CFA.

### Statistical methods

Statistical analyses were conducted in R v3.4.3 (R Development Core Team^64^), and included the R libraries ‘chron’, ‘doBy’, ‘nlme’, and ‘car’. Analyses of linear trends over the 10-years observation period were based on non-hierarchal generalized linear models (GLMs), with predictors based on date (to estimate the decadal trend), temperature, salinity and month (as chronological factor, to account for other seasonal changes such as irradiance or productivity). Since the focus of this study was on long-term trends rather than short-term fluctuations, the data were averaged within months. For NRSYON, the predictors also included dissolved nutrients (total silicate, total phosphorus, and dissolved inorganic nitrogen as the sum of nitrate and ammonium). Dissolved inorganic nitrogen was fourth-root transformed to approximate normal distribution, while the other variables approximated normal distribution. Estimates of trends in temperature and salinity were based on Gaussian GLMs using the decadal trend (date) and months as the predictors. Model effects were mostly additive, except when indicated. Partial dependence plots were used to show the effects of each predictor on the responses while holding other predictors constant at their mean values (or their respective categorical values)^10,65,66^. To provide comparison with more traditional non-statistical DIC, A_T_ and *f*CO_2_ data presentation, the trends analyses were also shown on salinity-normalised DIC and A_T_ values (DIC-S, A_T_-S) following Friis et al.^40^ (mean salinity GBRWIS: 35.49, NRSYON: 35.29), and on temperature normalised *f*CO_2_ (*f*CO_2_-T) following Takahashi et al.^39^ (mean seawater temperature GBRWIS: 24.34 °C, NRSYON: 25.81 °C). The results were almost identical to those from the non-hierarchal GLMs as the latter investigate the contribution of each factor while simultaneously controlling for the effects of all other factors included in the models.

We hindcasted the carbonate chemistry of the two data series to 1958, based on the assumption that GBR A_T_ values, and the slopes and offsets between our GBR data and the data from Mauna Loa since 1958 (www.esrl.noaa.gov/gmd/ccgg/trends/) have remained similar. To do so, we calculated the slopes and offsets for the current (2009–2019) mean *f*CO_2_ winter minima and summer maxima for both stations and the annual mean Mauna Loa (Table 1), and used these offsets to approximate past *f*CO_2_ envelopes at the stations.

## Supplementary information

Supplementary Information
