# Supplementary material for: Progressive seawater acidification on the Great Barrier Reef continental shelf
Source: Sci Rep. 2020 Oct 27;10:18602. doi: 10.1038/s41598-020-75293-1 (PMC7592051; doi:10.1038/s41598-020-75293-1)
Supplement: Supplementary file 1 — Supplementary Information [file 41598_2020_75293_MOESM1_ESM.docx]

Supplementary Material: Progressive seawater acidification
on the Great Barrier Reef continental shelf

Katharina E. Fabricius ^1 ,*^, Craig Neill ^2^, Erik Van Ooijen ^2^, Joy N. Smith ^1^, Bronte Tilbrook ^2,3^

1 Australian Institute of Marine Science, PMB 3, Townsville Qld 4810, Australia

2 CSIRO Oceans and Atmosphere, Castray Esplanade, Battery Point 7004, Australia

3 Australian Antarctic Program Partnership, University of Tasmania, Hobart 7001 Australia

* Corresponding author: [k.fabricius@aims.gov.au](mailto:k.fabricius@aims.gov.au)

**Table S1:** NRSYON: Environmental and temporal factors associated with changes in temperature and salinity normalised seawater carbon chemistry variables. Non-hierarchal generalized linear model (GLM) analysis of monthly depth-averaged data (N = 107 months). Tabled are the decadal trends (Date) and seasonality (Month) and nutrients as predictors for salinity normalised^1^ dissolved inorganic carbon (DIC-S) and total alkalinity (A_T_-S), and temperature-normalised^2^ fCO_2_ (fCO_2_-T) (Fig. 6, Supplementary Fig. S1c).

| (b) |  | DIC-S | | A_T_-S | | *f*CO_2_-T | |
| --- | --- | --- | --- | --- | --- | --- | --- |
|  | df | F | P | F | P | F | P |
| Decadal trend | 1 | 11.24 | 0.001 | 0.139 | 0.710 | 11.60 | <0.001 |
| Month | 11 | 4.459 | <0.001 | 3.848 | <0.001 | 1.905 | 0.049 |
| Temperature | 1 | 2.237 | 0.138 | 6.621 | 0.012 | 24.98 | <0.001 |
| Salinity | 1 | 7.629 | 0.007 | 3.737 | 0.056 | 4.944 | 0.029 |
| DIN | 1 | 7.113 | 0.009 | 4.144 | 0.045 | 0.937 | 0.336 |
| Phosphate | 1 | 4.842 | 0.030 | 1.142 | 0.288 | 2.291 | 0.134 |
| Silicate | 1 | 0.998 | 0.321 | 4.602 | 0.035 | 10.91 | 0.001 |

**
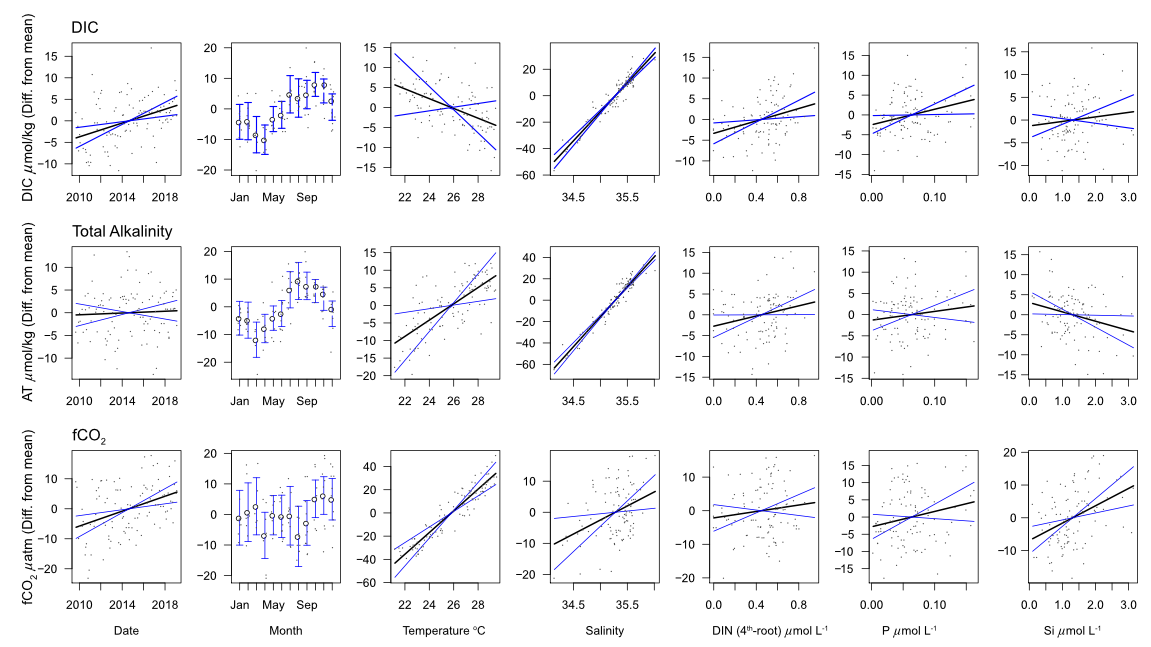
Supplementary Figure S1:** NRSYON: Partial effects plots of changes over time, across months, with temperature, salinity and nutrients, in the depth-averaged seawater concentrations of (a) DIC and A_T_ (measured), and calculated fCO_2.;_ (b) the other calculated carbon chemistry variables, (c ) responses of salinity normalised^1^ DIC and AT (DIC-S, AT-S), and temperature normalised^2^ fCO_2_ (fCO_2_ -T). Values on the y-xis represent differences from the mean values (Table S1), solid thick lines show the trends, thin lines are 2 SE, and grey dots the residuals.

**Supplementary Figure S1b**

**
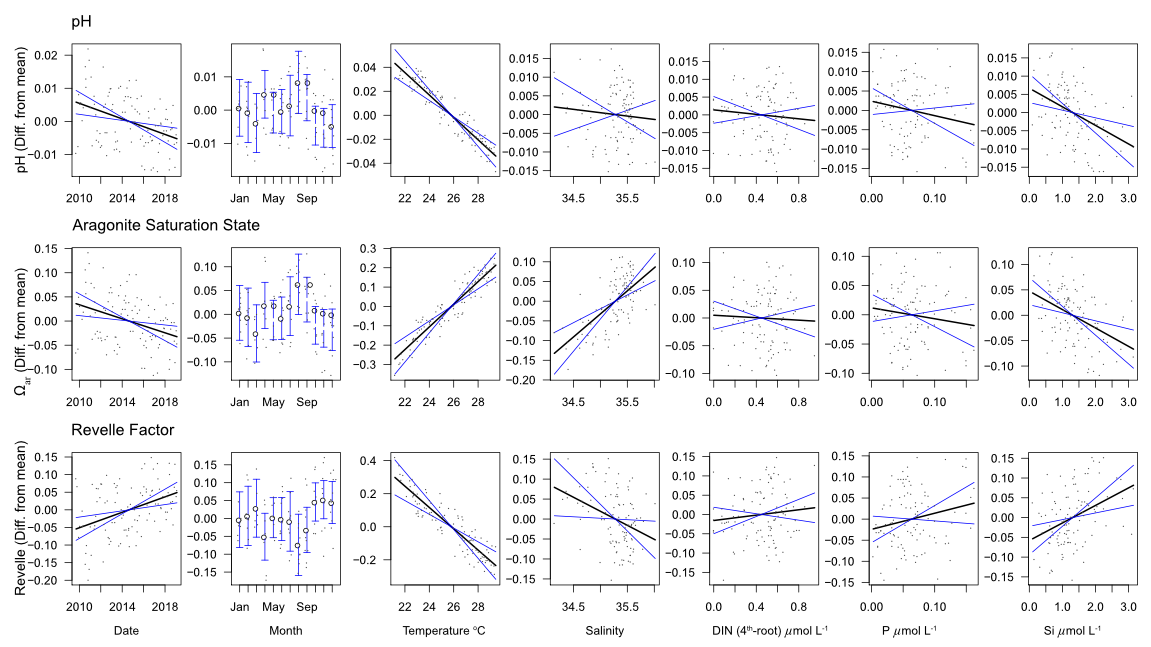
**

**Supplementary Figure S1c**

**
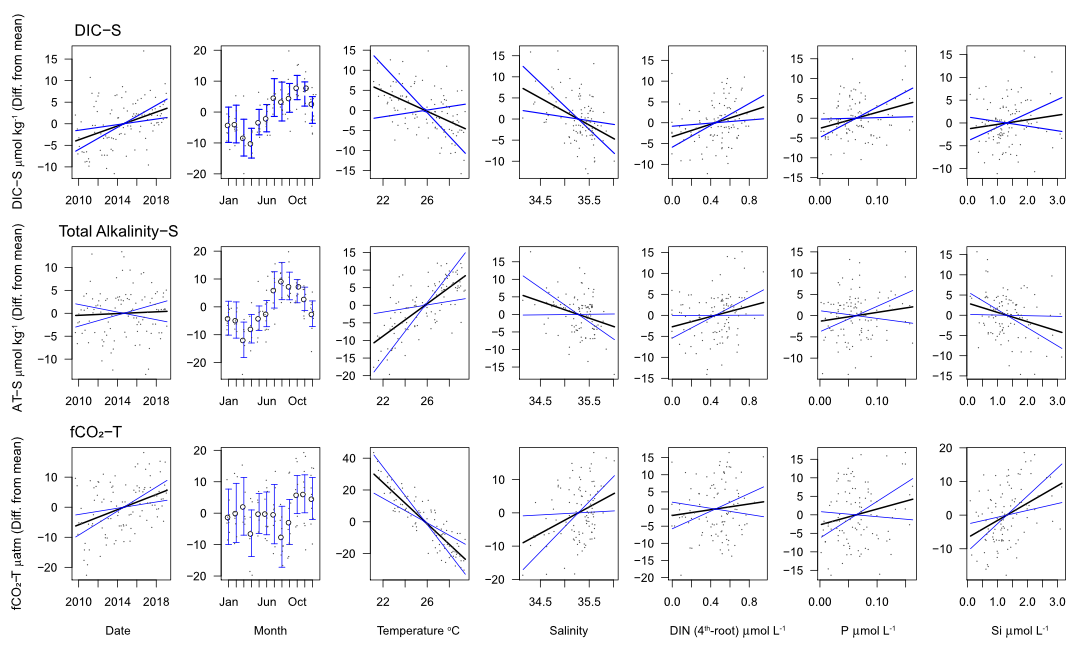
**

1 Friis, K., Körtzinger, A. & Wallace, D. W. R. The salinity normalization of marine inorganic carbon chemistry data. *Geophysical Research Letters* **30**, 2002GL015898, doi:10.1029/2002gl015898 (2003).

2 Takahashi, T. *et al.* Global sea-air pCO2 flux based on climatological surface ocean pCO2, and seasonal biological and temperature effects. *Deep Sea Research Part II: Topical Studies in Oceanography* **49**, 1601-1622, doi:10.1016/S0967-0645(02)00003-6 (2002).
